# Supplementary figures and images for: Cloning and Characterization of a Novel N-Acetyl-D-galactosamine-4-O-sulfate Sulfatase, SulA1, from a Marine Arthrobacter Strain
Source: Mar Drugs. 2024 Feb 23;22(3):104. doi: 10.3390/md22030104 (PMC10972148; doi:10.3390/md22030104)

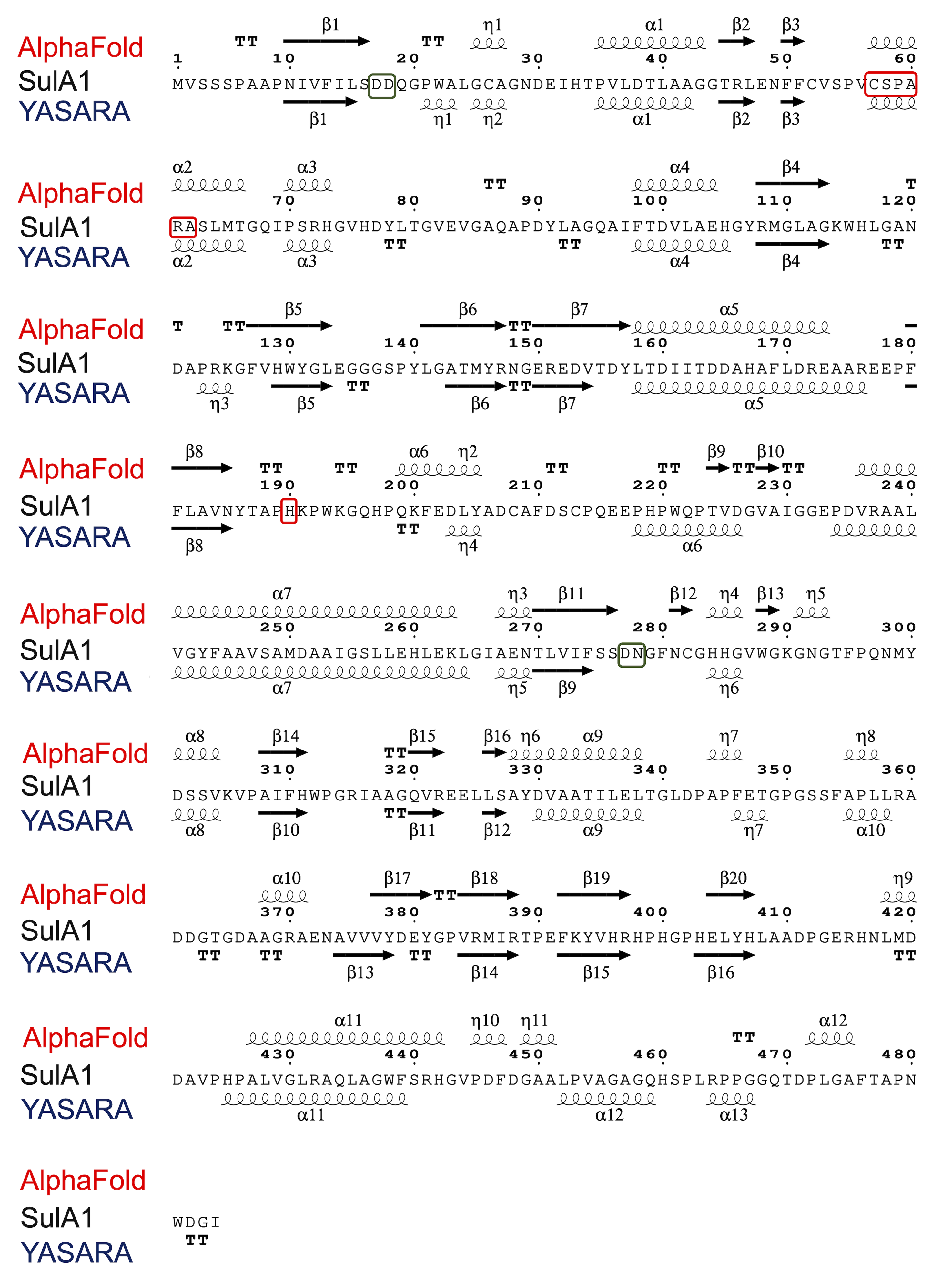

Supplement: Supplementary file 1 [file marinedrugs-22-00104-s001.zip › S1.tif]

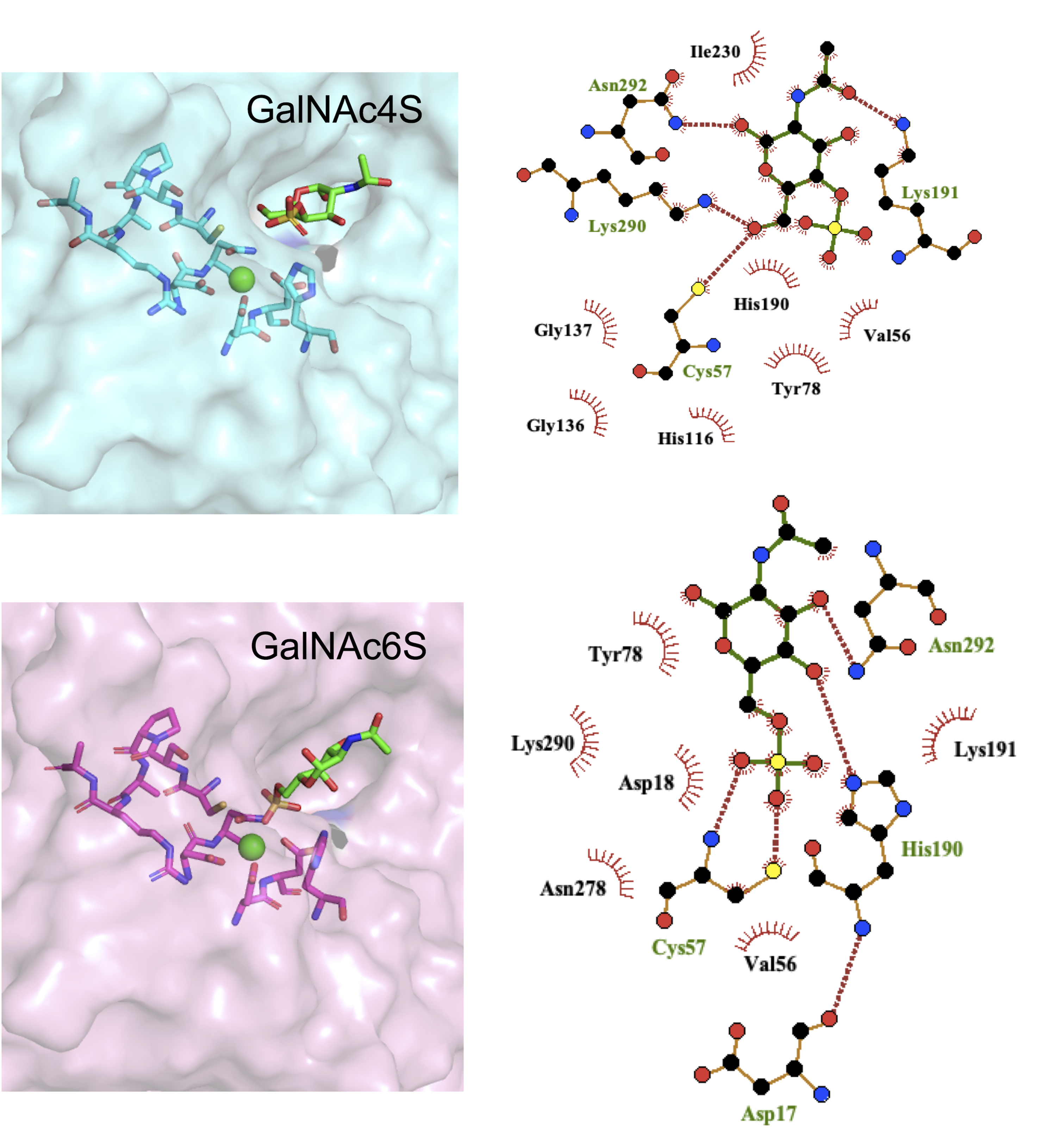

Supplement: Supplementary file 1 [file marinedrugs-22-00104-s001.zip › S2.tif]

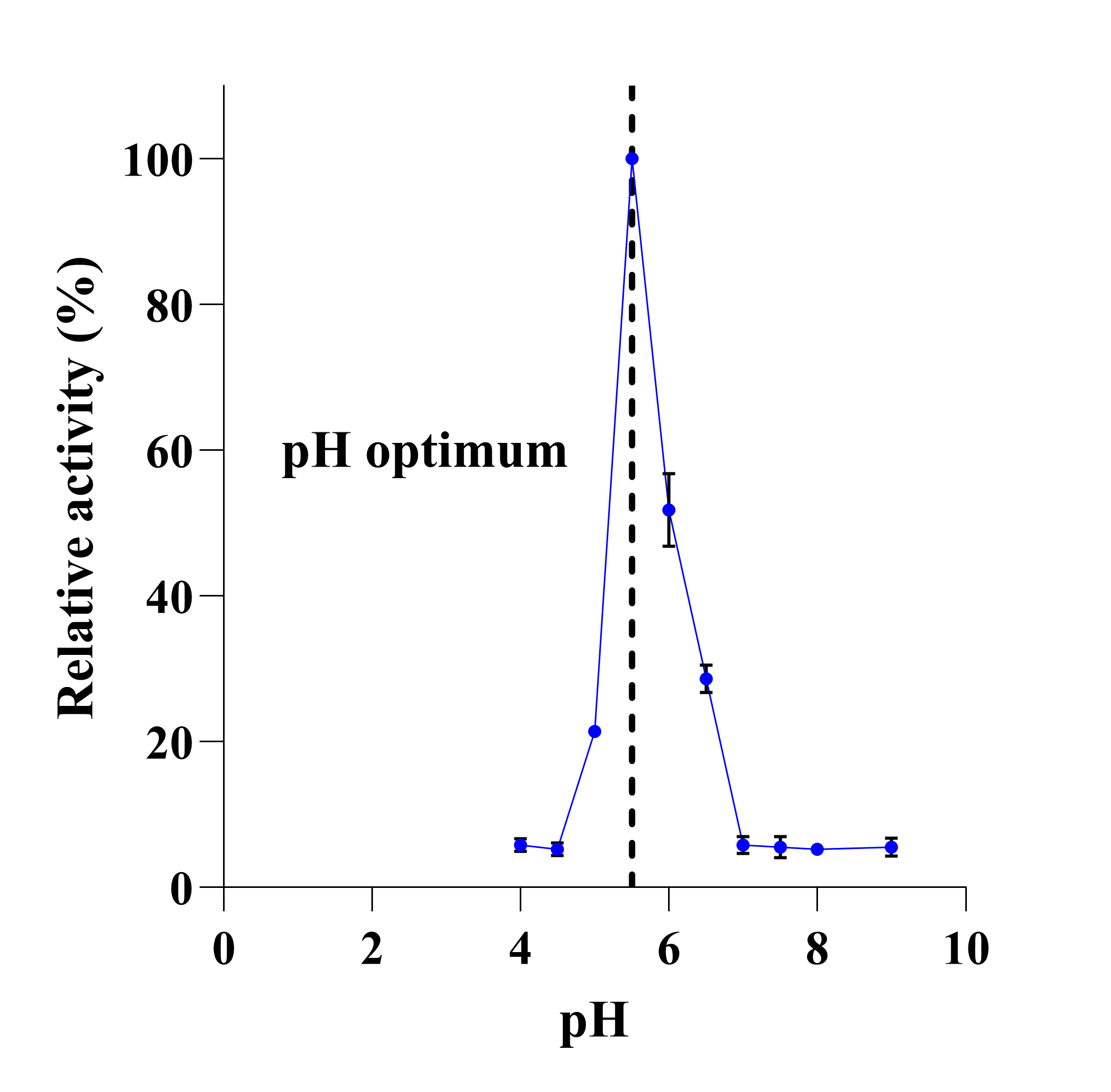

Supplement: Supplementary file 1 [file marinedrugs-22-00104-s001.zip › S3A.tif]

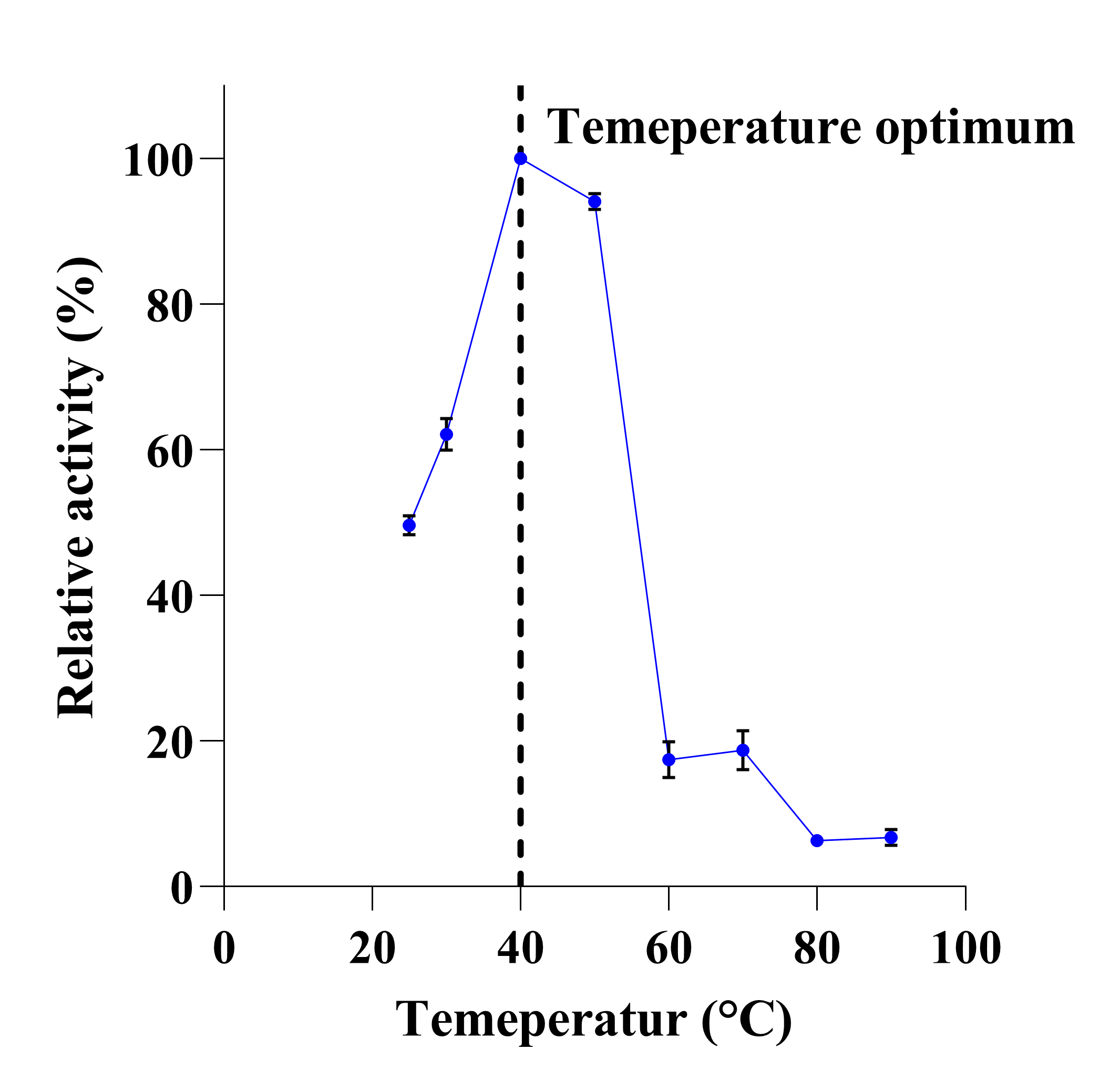

Supplement: Supplementary file 1 [file marinedrugs-22-00104-s001.zip › S3B.tif]

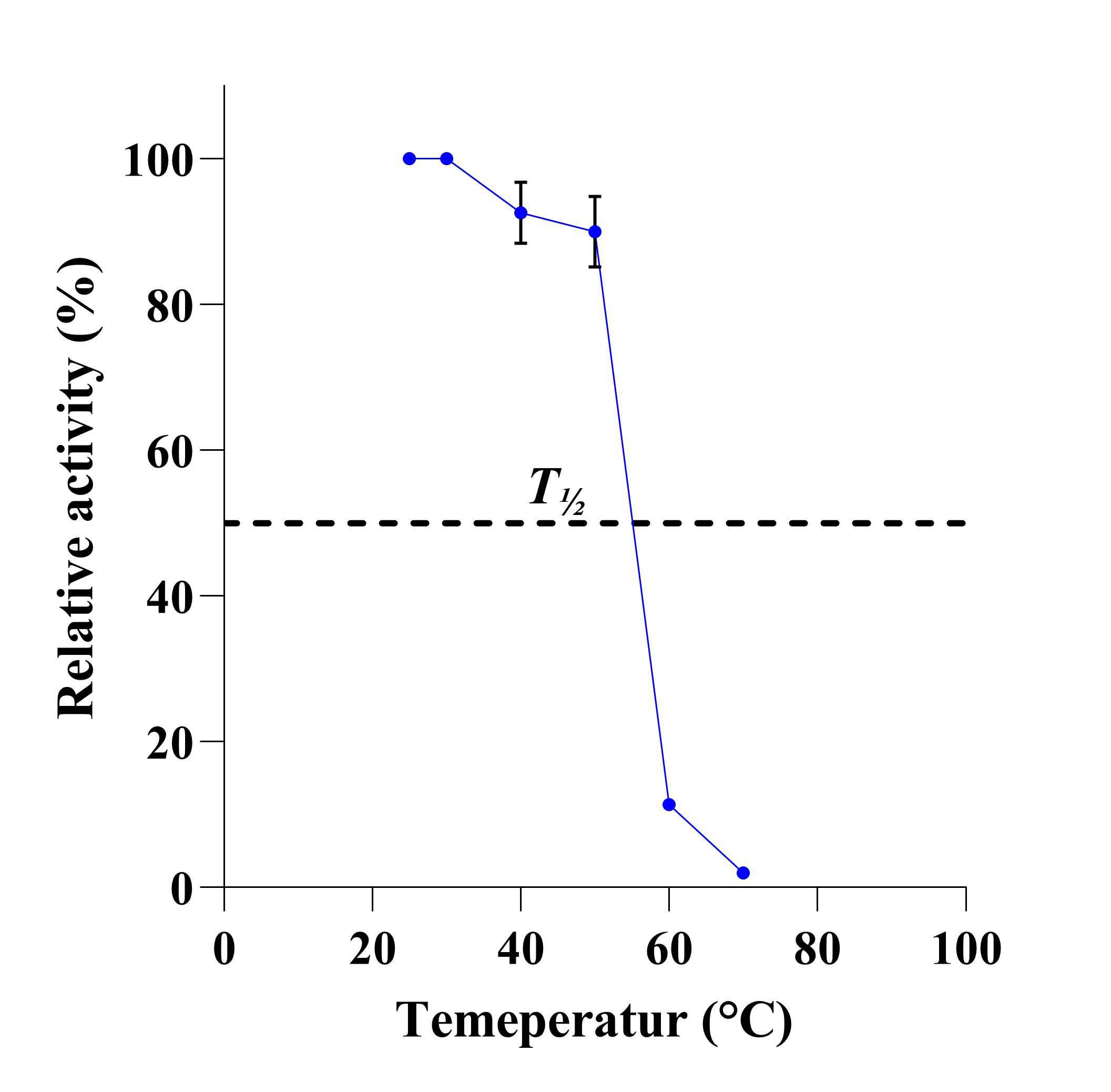

Supplement: Supplementary file 1 [file marinedrugs-22-00104-s001.zip › S3C.tif]

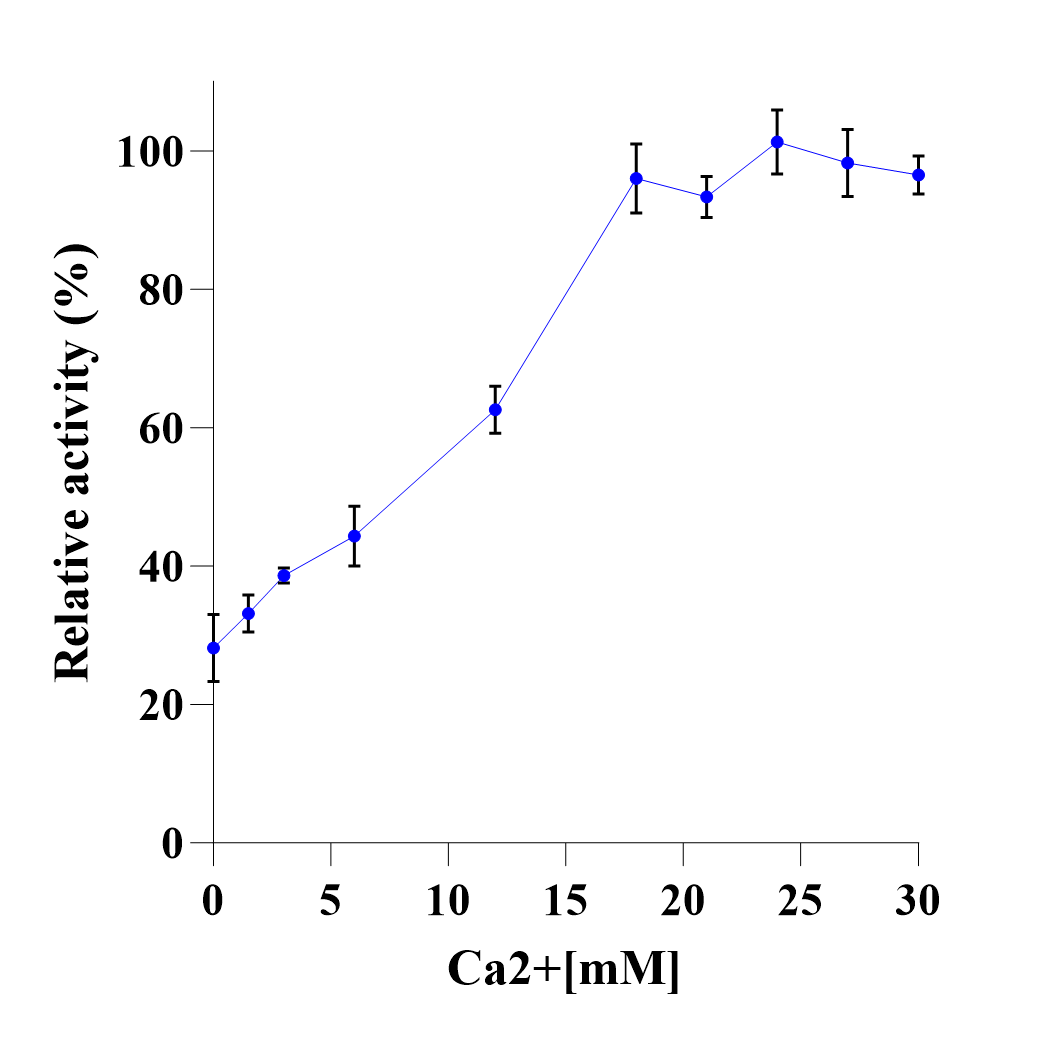

Supplement: Supplementary file 1 [file marinedrugs-22-00104-s001.zip › S3D.tif]

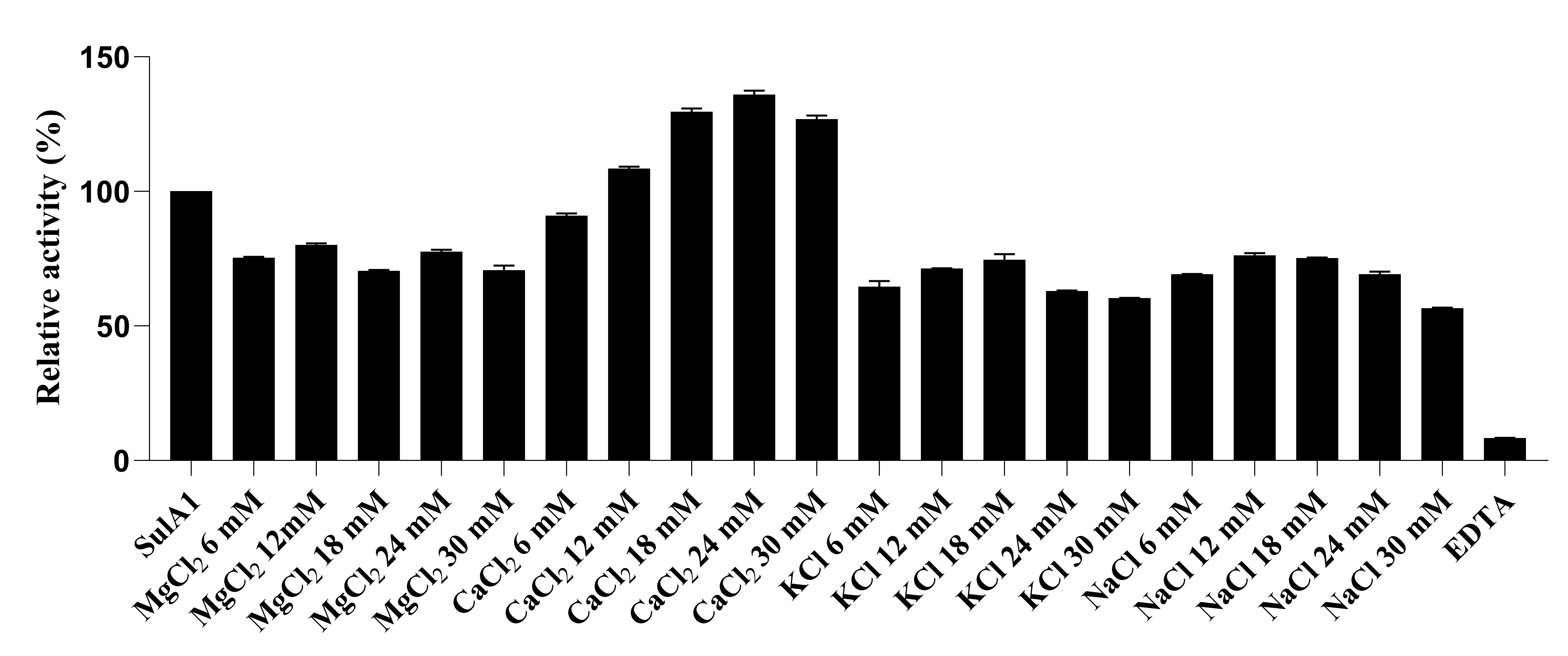

Supplement: Supplementary file 1 [file marinedrugs-22-00104-s001.zip › S4.tif]
